# Partial reversal of color vision impairment in type 2 diabetes associated with obstructive sleep apnea

**DOI:** 10.3205/oc000087

**Published:** 2018-03-08

**Authors:** Rajiv Raman, Aditya Verma, Sangeetha Srinivasan, Deepak Bhojwani

**Affiliations:** 1 Shri Bhagwan Mahavir Vitreoretinal services, Sankara Nethralaya, Chennai, India

**Keywords:** diabetes mellitus, tritan color defect, obstructive sleep apnea, continuous positive airway pressure ventilation

## Abstract

**Objective:** Tritan anomaly is a known acquired color defect seen in diabetic patients, with or without the evidence of clinical signs of diabetic retinopathy (DR). We report a case of a 45-year-old diabetic patient with tritan pattern color defect associated with obstructive sleep apnea and its partial reversal with continuous positive airway pressure (CPAP) ventilation.

**Methods:** A 45-year-old male with diabetes, wildlife photographer by profession, presented with specific complaints of seeing all objects in the surrounding with a greenish tinge in both the eyes. He underwent a comprehensive eye examination including Farnsworth-Munsel 100 (FM 100) hue test, multifocal electroretinogram, microperimetry, spectral domain optical coherence tomography (SDOCT), and arterial oxygen saturation.

**Results:** The subject was found to have a low arterial oxygen saturation (PaO2) of 86%. He was then advised inhalation of 100% oxygen for 15 min, following which he reported improvement in his visual symptoms. FM 100, OCT, and microperimetry were repeated after oxygenation. He was referred to a specialty hospital for further evaluation of the cause for reduced blood oxygen saturation and was further advised for sleep study, where he was diagnosed to have obstructive sleep apnea (OSA) with an apnea-hypopnea index of 20.9.

**Conclusion:** The subject was advised weight loss measures and oral application of continuous positive airway pressure. Since then, he is under our regular follow-up and has never experienced or complained of any color vision problems. This case report highlights the presence of associated systemic disorders like obstructive sleep apnea in individuals with diabetes that can present with color vision problems.

## Introduction

Type 2 diabetes is a major public health problem concerning high morbidity, mortality, and health-care costs. Recent research demonstrates the likelihood of a relationship between type 2 diabetes and obstructive sleep apnea. Obstructive sleep apnea (OSA) is the most common form of sleep disordered breathing [1] accounting for over 80% of the sleep apnea cases. Estimates suggest that up to 40% of people with OSA have diabetes, but the incidence of new diabetes in people with OSA is not known [2]. 

Tritan-like acquired color vision defects have been reported to occur in diabetes. Dean et al. [3] demonstrated a partial reversal of protan and tritan color defects with inhaled oxygen in insulin-dependent diabetes mellitus. 

We report a case of type 2 diabetes with tritan color vision defect that showed partial reversal of his color vision defect with inhalation of oxygen and was also found to have moderate obstructive sleep apnea. 

## Case description

A 45-year-old male, wildlife photographer by profession, presented with specific complaints of seeing all objects in the surrounding with a greenish tinge in both the eyes for a month. He was diagnosed with diabetes 5 years ago and was prescribed injectable human insulin 12 IU (international units) by his treating physician. His past diabetic control as recorded by his physician was excellent (HbA_1c_=6.8%) with no secondary systemic complications attributable to diabetes. His previous drug history regarding the usage of oral hypoglycaemic agents, addictions or other drugs for any major systemic illness was negative. On examination, his best corrected visual acuity was 20/20 and N6 in both eyes. His anterior and posterior segment examination were unremarkable. Intraocular pressure was 14 mm Hg in his right eye and 12 mm Hg in his left eye.

**Figure 1:**
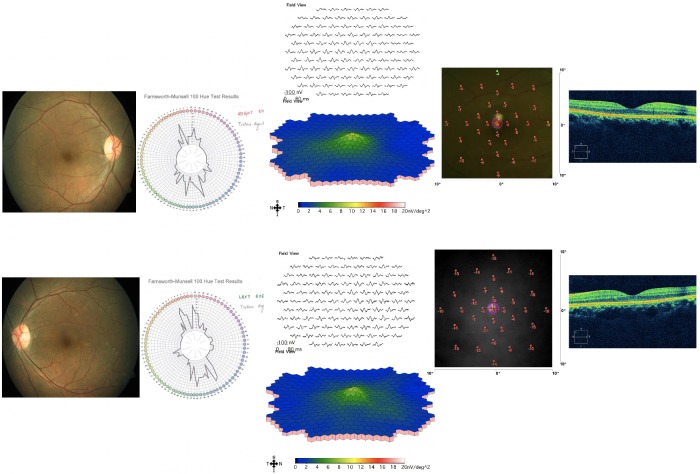
Fundus photograph, Farnsworth-Munsell 100 hue test, multifocal electroretinogram result, microperimetry and optical coherence tomography of both eyes of the patient

**Figure 2:**
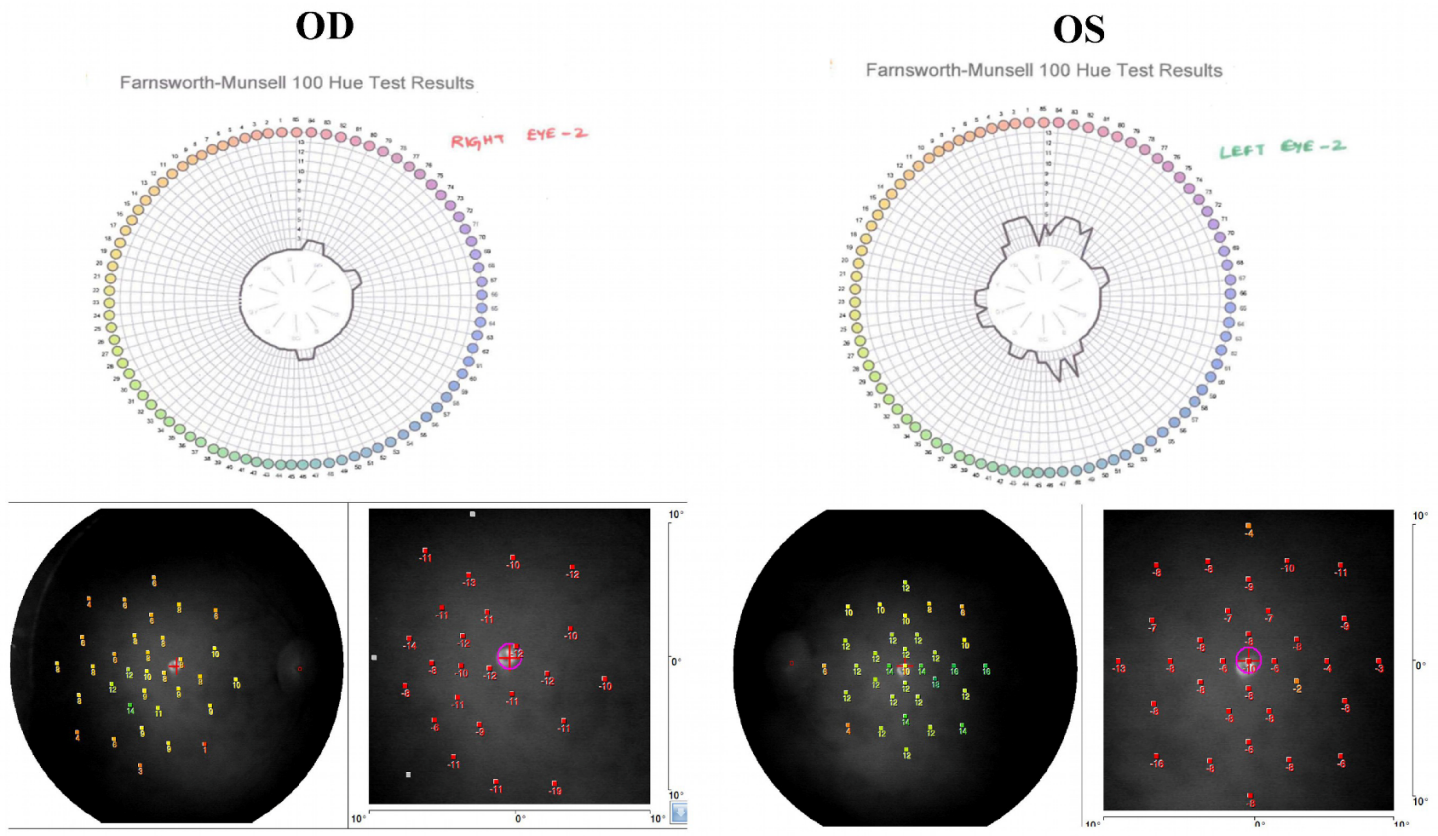
Post-oxygenation therapy; Farnsworth-Munsell 100 hue test and microperimetry result

Fifty-degree posterior pole retinal photographs were obtained with the Carl Zeiss FF 450 Plus IR Fundus Camera for documentation. The Farnsworth-Munsell 100 hue test (Munsell Color Services Laboratory X-Rite Inc, Kentwood, MI, USA) showed a total error score (TES) of 144 and 172 in right and left eye, respectively, with a tritan pattern (Figure 1 (Fig. 1)). Both values were above 100, indicating a low color discrimination. Multifocal electroretinogram (Electro-Diagnostic Imaging Inc, Veris™ Science V 5.2.2X, San Francisco, CA, USA) showed reduced cone responses. Microperimetry (MP 1, Nidek Instruments Inc, Padova, Italy) of both eyes showed reduced retinal sensitivities throughout the macular area with a mean retinal sensitivity of 13.2 dB and 9.2 dB in his right and left eye, respectively. Spectral domain optical coherence tomography (OCT) (Cirrus OCT, Carl Zeiss Meditec, Dublin, CA, USA) of both eyes showed normal foveal architecture with foveal thickness of 172 and 175 microns in his right and left eye, respectively (Figure 1 (Fig. 1)). His arterial oxygen saturation (PaO2) documented on arterial blood gas (ABG) was 86%. Other parameters like pH, PaCO2, H+, HCO3– were within normal limits. On inhalation of 100% oxygen for 15 min, he reported improvement in his visual symptoms. We repeated FM 100 hue test, which showed a TES of 12 and 72 in his right and left eye, respectively, indicating a superior color vision discrimination in his right eye and an average color discrimination in his left eye. The mean retinal sensitivity with microperimetry was 13 dB and 11.6 dB in his right and left eye, respectively (Figure 2 (Fig. 2)). He was referred to a specialty hospital for further evaluation of the cause for reduced blood oxygen saturation. His cardiac evaluation (electrocardiogram, 2D echocardiogram), pulmonary function tests (including chest X-ray), and blood investigations (hemogram, liver and kidney function test) were within normal limits. Based on the history of snoring for the past 2 years and obesity (body mass index 32 kg/m^2^), the physician advised him to undergo overnight four channel sleep study. The sleep study suggested moderately severe sleep disordered breathing with an apnea hypopnea index of 20.9. He was advised weight loss measures and oral application of continuous positive airway pressure (CPAP). Since then, he is under our regular follow-up. He never experienced or complained of any color vision problems.

## Discussion

Color vision deficits observed in non-diabetic individuals in association with reduced arterial oxygen saturation in high altitudes are reversible when the subjects come to normal altitude of habitation or when oxygen supplementation is provided [4]. In this case study, we observed that color vision deficits in the presence of lower blood oxygen levels improved with oxygen inhalation in a patient with diabetes who was diagnosed with obstructive sleep apnea. We believe that the blue-yellow color deficit is more likely due to lower blood oxygen and not attributable to well-controlled diabetes. We provide several likely explanations for our proposition. 

For instance, in patients with type 2 diabetes, the tritan thresholds positively correlate with the diabetes duration, but not with the age of the patient, suggesting that the tritan deficiency is related to the underlying pathological changes in diabetes [5]. 

Patients with exudative maculopathy are expected to demonstrate marked defects of color vision, wherein the central macular thickness on OCT is above 260 microns [6]. In our case, the central macular thicknesses were 172 microns in the right eye and 175 microns in the left eye. Since the study patient did not exhibit clinical signs of DR or maculopathy, this is unlikely to be related to our study outcome. 

Media opacities such as brown cataracts, haemorrhage (vitreous or intraretinal or subretinal) filter shorter wavelengths of visible spectrum of light and may cause blue-yellow color vision deficits, but are not expected to improve with oxygenation. Only 9% of cones are sensitive to blue spectrum. Even if it is assumed that in diabetes, there is a generalized or equal loss of all types of cones, the matrix of S-cones (blue spectrum) is more disturbed as compared to L- (red spectrum) and M- (green spectrum) cones [7] and is more susceptible to oxidative stress [7]. Nevertheless, we observed an improvement in color vision in our study subject after inhalation of 100% oxygen. Deficits in color vision in diabetic patients may be associated with reduced retinal arterial oxygen saturation or oxygen tension in the tissue [3]. Brandl and Lachenmayr [8] reported that retinal function is sensitive to blood oxygenation. Some of the ophthalmic manifestations of reduced blood oxygenation include impairment of color vision, visual acuity, and changes in retinal sensitivity under photopic conditions. We observed an improvement in the patient’s color vision with inhaled oxygen [3]. He was advised weight loss measures and CPAP. He never experienced color vision problems to date. Therefore, our study findings appear to be in favour of lower oxygen saturation as observed in obstructive sleep apnea. 

## Conclusions

This case report highlights the need to keep in mind the presence of OSA in diabetic patients who can present with color vision problems in the absence of clinical signs of DR.

## Notes

### Competing interests

The authors declare that they have no competing interests.
